# Irrepressible: An Interview with Mark Ptashne

**DOI:** 10.1371/journal.pgen.1005351

**Published:** 2015-07-16

**Authors:** Jane Gitschier

**Affiliations:** Departments of Medicine and Pediatrics and Institute for Human Genetics, University of California San Francisco, San Francisco, California, United States of America

**Fig 1:**
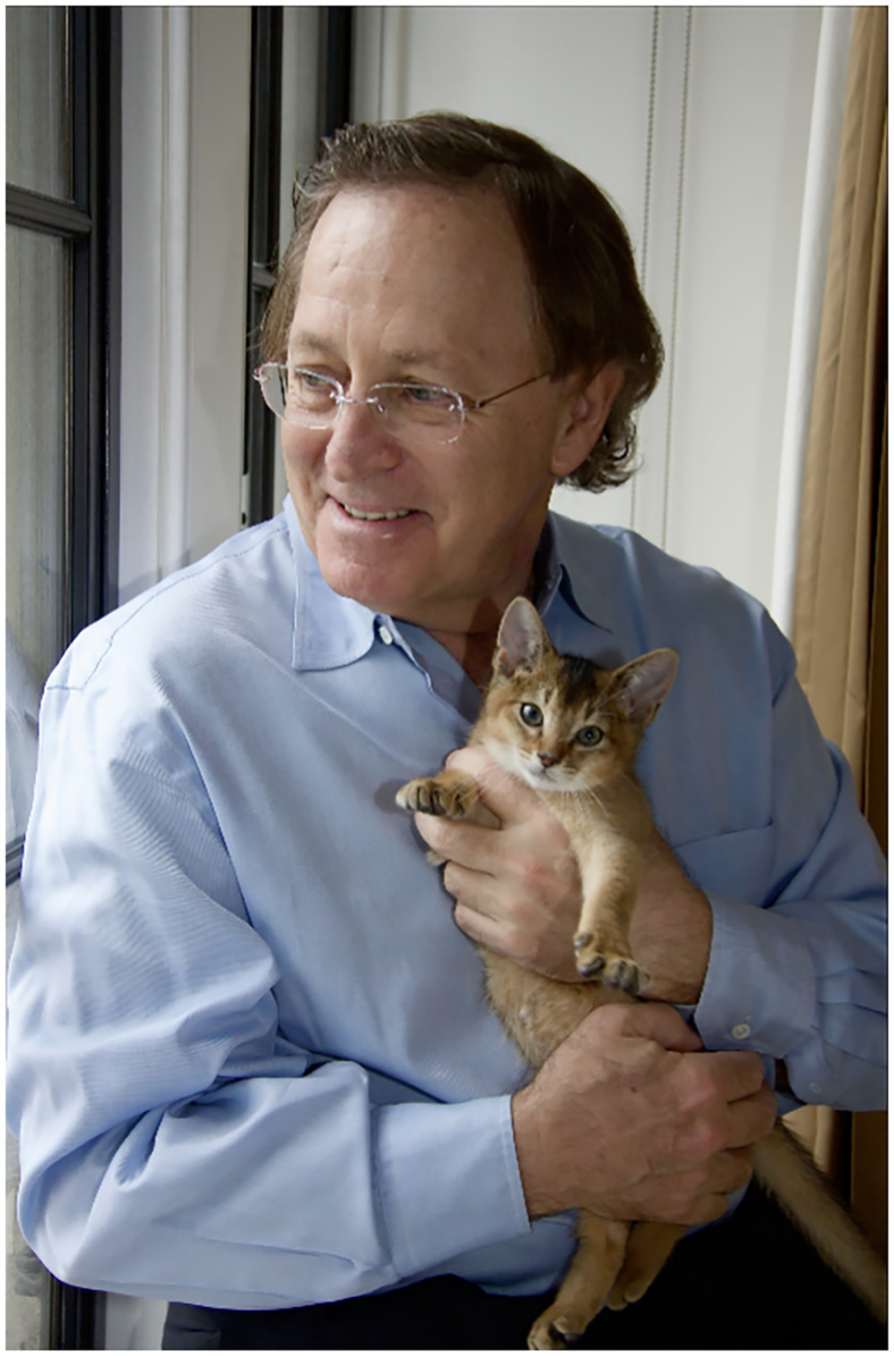
Mark Ptashne and McCoy. Image courtesy of Mark Ptashne.

While winter storms crippled almost every state from Wisconsin to Florida, our own force of nature blew into California in January in the compact human form of Mark Ptashne (Fig 1). Mark, who holds the Ludwig Chair of Molecular Biology at Sloan-Kettering in New York, had arrived to give a series of seminars on the West Coast. And if that weren’t enough, he also came prepared for a soirée, with his violin in tow.

In the late 1960s, while still a Junior Fellow at Harvard, Ptashne became known for the isolation of a specific protein—the bacteriophage λ repressor—and the demonstration that it could bind to specific sequences of double-stranded DNA called operators. This experiment, together with the isolation of the lac repressor by his colleague Walter Gilbert, made manifest the holy grail of that time: François Jacob and Jacques Monod’s elusive repressors, predicted by genetic evidence but working through unknown mechanisms. Over the ensuing decades, Ptashne and his laboratory not only clarified gene regulation in molecular detail in λ, they extended these profound discoveries from bacteria to eukaryotes, making it possible to think about development and evolution rationally, in molecular terms. With his sturdy keel and breakneck speed, the strong wake of Ptashne’s scientific career is impressive. One colleague, quoted in a New York Times article, commented, “Everything we know about gene transcription has come from...Ptashne.” Ptashne is highly regarded for his clarity of thought, as epitomized by his book *A Genetic Switch* and another, *Genes and Signals*, co-authored by Alexander Gann. His laboratory’s recent work on gene regulation in eukaryotes, including analysis of sequence specificity and occupancy of nucleosomes, has led to his commentaries on the misplaced efforts of much current epigenetic research.

I had been pestering Mark to do a *PLOS Genetics* interview, and for some time he managed to wriggle out of it. But late on a Saturday afternoon, I was summoned to the home of Sandy Johnson, Mark’s former thesis student and, as it happens, my former husband. Mark was ready to chat, and asked, by the way, could I please bring some Epsom salts so he could soak his hand before the concert. Johnson was called on occasionally to interpret, clarify, and generally calm things down at several points in the interview. When the dust settled, I was left with a transcript of a spirited but highly disjointed conversation that, when redacted, went something like this:

**Gitschier:** I want to talk to you about your upbringing and then I want to talk to you about the big repressor time period of your life. Let’s just start at the beginning. What city were you born in?

**Ptashne:** Chicago, Illinois.

**Gitschier:** Now, your parents. What were their names?

**Ptashne:** Fred and Millie.

**Gitschier:** OK, and what were their professions?

**Ptashne:** Well, my father was a businessman of sorts. When I was about ten, we moved to Minneapolis so he could be in my uncle’s snowsuit business.

**Gitschier:** Really?

**Ptashne:** Yes! Isn’t that weird? Uncle Nate. It was cheaper to make snowsuits in Minneapolis than Chicago. Then there was a candy company after the snowsuit thing. My mother was a social worker and then, I think, a travel agent. But they remained associated with the “Progressive Movement.”

**Gitschier:** So your parents...

**Ptashne:** They were lefties.

**Gitschier:** Were they born in the United States?

**Ptashne:** Yes.

**Gitschier:** And they were both Jewish.

**Ptashne:** Oh, for God’s sake, yes. If we’re talking about a leftist in the ‘30s and ‘40s, it’s a package deal.

**Gitschier:** So, tell me more!

**Ptashne:** One good thing was that being lefties we could go down to Mexico to visit the famous Cedric Belfrage, the editor of *The National Guardian* [a left-wing paper]. And another good thing was that Paul Robeson would come to the house and sing “Water Boy” as I sat under the piano. Wow.

**Gitschier:** Paul Robeson!

**Ptashne:** I used to think that my parents’ political bent gave me an advantage in science.

**Gitschier:** Why?

**Ptashne:** Because looking back on it, when you have a firm ideology and you never actually have to read anything—you never read Marx or anything—still you always *knew*. Most of what you heard was false, but there always was an underlying truth! Which is the basic stance of Nietzsche and Freud and so on. Don’t believe anybody. So, like Moby Dick, and unlike the hapless Ahab who was forced to skim the surfaces, you were able to dive into the depths to find that truth. (I’m stealing this line from Joe Paterno.)

Put another way, a skeptic is someone who can imagine a better answer. In retrospect, all this was good training.

So then, we had trouble after the 20th Congress [1956] when Khrushchev denounced Stalin. My parents’ ideological world collapsed. Suddenly they lost interest in Russia and became very good friends with China! It’s amazing how they pulled this off. My mother arranged for Minneapolis to become a sister city with some city in China, and they took tours to China.

A memorable event for me was going to Cuba, just after Tet [the North Vietnamese offensive in early 1968]. That’s where I met Fidel.

**Gitschier:** You did?

**Ptashne:** Of course! We chopped a little sugar cane and lay around the pool chatting up the American lady lefties.

**Gitschier:** Why did you go to Cuba? We’ll need to back up, but go ahead and answer that question and we can rewind.

**Ptashne:** Fidel convened something called the Cultural Congress of Havana. And this was to bring all the great cultural figures to Havana to discuss...something. The science that the South Americans have a history in is neurology. I had been working in a laboratory of Frank Morrell, a neurologist at the University of Minnesota. In fact, my first published paper was on the effect of diphenylhydantoin on peripheral nerve transmission in epileptogenic... and the second paper was something equally...[obscure].

Frank was a good friend of my parents.

**Gitschier:** Maybe you worked there for a summer during college?

**Ptashne:** Yes. Now Frank met an unhappy ending. He ended up with a huge scandal about a fabricated paper, which is the most amazing thing, because Frank was one of these guys who was just fanatical about the data.

**Gitschier:** OK, Cuba. Somehow you related Cuba to your working with Frank Morrell. Did you get invited there because of your fabulous research with him?

**Ptashne:** No, because the scientists they invited included not Frank, it turns out, but a neurologist at Columbia who worked on something called evoked potentials, and Frank said, “I’ll get you included in this group.”

**Gitschier:** So you wanted to go to Cuba?

**Ptashne:** Well, of course I did!

**Gitschier:** Why?

**Ptashne:** Are you kidding? Every major figure on the American left was there, and the added frisson was that it was technically illegal to go to Cuba. But a Supreme Court decision had come down that said passports couldn’t be revoked for going where you weren’t supposed to go.

**Gitschier:** Who else was there?

**Ptashne:** Tom Hayden, David Dellinger, Jules Feiffer, Bob Scheer. Just everybody. The greatest thing was meeting Jules Feiffer. See, you would go to Mexico and sit in some Embassy for a couple of days in order to get to Cuba. And that is where I met Jules. And we became fast friends and still are friends.

**Gitschier:** This seems like it was a transformational experience.

**Ptashne:** Yes!

**Gitschier:** Why?

**Ptashne:** Well first of all, Cubans are wonderful people. And I remember I was astounded to see a society where there were no billboards! It was a pure socialist thing, very romantic.

I remained a bona fide lefty until years later when I broke with the left over recombinant DNA. They said we should oppose the experiments because they were dangerous—mobilizing the masses and all that. Trouble was that it wasn’t true.

**Gitschier:** OK, now you’re jumping ahead to the ‘70s, and we need to go back *way* before ‘68 now.

**Ptashne:** I should tell you about going to Crested Butte, Montana [actually, Colorado].

**Gitschier:** OK, tell me!

**Ptashne:** At Reed College, in Portland, Oregon, where I was an undergraduate, we had a spellbinding genetics professor named Tahir Rizki, an Indian. And the great thing about him was he kept talking about the reciprocal crosses, and his eyes would twinkle!

**Gitschier:** What organism are we talking about?

**Ptashne:** *Drosophila*. I then got to spend my senior-year summer with the great Ed Novitsky. One thing I regret is that I never went back and contacted Ed again because he recently died. He wrote a little book not too long ago [*Sturtevant and Dobzhansky*, *Two Scientists at Odds*]. He was an intelligent, dry, witty character.

**Gitschier:** Where was Novitsky?

**Ptashne:** At the University of Oregon, in Eugene. Every summer he would go to Crested Butte and all the major fly people were there. Bruce Baker, Charles Remington the butterfly guy, and so on. And then one summer—I must have gone two years—H. J. Muller himself came. That was something. I was awestruck by this tiny giant. To get an idea of what he did, read James Schwartz’s marvelous book *Pursuit of the Gene* [and check out the *PLOS Genetics* interview with Schwartz].

**Gitschier:** So Crested Butte—I take it there is a lab there?

**Ptashne:** Now there is. It used to be argued, “My God, you’re going to put electric lights in Crested Butte, and pretty soon there’ll be sidewalks!”

It was a famous fly lab. The Drosophilists would go there for the summer and do wonderfully complicated experiments. Have you read my paper about strong and weak centromeres at the second anaphase of *Drosophila melanogaster*?

**Gitschier:** I think I must have missed that. This is the work that you did with Ed Novitsky at Crested Butte?

**Ptashne:** Yes.

Then he did a neat trick. Molecular biology was just coming up at Eugene, and the new center there was headed by Aaron Novick and Frank Stahl. Ed despised them [because they were molecular biologists], or so he said. He recommended I go there. I’m not sure why.

And I did spend a summer with Aaron and Frank and they were big influences. Aaron would say things like, “You have to go to meetings, because it’s only by looking at the guy that you can tell whether to believe him.” It’s hopeless now because there are too many guys and too many meetings, and of course, they’re not going to invite me!

The point here is that the only people who know experiments in depth are those who have done them and are reporting them, and you have to have some way to guess as to how hard that person has challenged himself or herself to get it right. Scientists differ in the degree to which they challenge themselves. Remember Nietzsche: “The problem is not fooling others, it’s fooling oneself.”

And Frank had all kinds of good stuff, too. He used to say, “Most of the time you are rehearsing to do the experiment, and then you finally do it.”

And I managed to do exactly what they hoped: I disproved Jacob and Monod! So they were thrilled! But what I had actually done was mix up the tubes!

**Gitschier:** Oh come on, are you serious?

**Ptashne:** Jacob and Monod had by then become my heroes. Aaron had spent time at the [Institut] Pasteur, and as much as he adored Jacob, he wanted to get them on something. I remember they [Novick and Stahl] were so excited by my results! But we soon found out they were fictitious. You cannot realize how easy it is to fool yourself until you do experiments, even if you don’t mix up the tubes! That’s why you need friends who will constantly challenge you.

**Gitschier:** So this was your claim to fame, the summer of ‘61—you mixed up the tubes.

**Ptashne:** And then it was Frank—this is Matt’s [Meselson] story—you’ll have to get Matt to tell it...

**Gitschier:** Yeah, but I have *you* here.

**Ptashne:** At that point Matt and Frank had some tiny falling out.

**Gitschier:** This was after the Meselson-Stahl experiment?

**Ptashne:** Yes.

**Gitschier:** Where was that experiment done?

**Ptashne:** Caltech. And Matt stayed at Caltech.

And so in order to somehow slow down Matt, Frank decided to send me as Matt’s graduate student! That’s when Frank said, “We’ve got to send this guy (i.e., me) to Meselson!” It was a Trojan horse sort of thing.

So I called Matt and went to visit him, and just at that point, Jim [Watson] convinced Matt to move to Harvard. So we ended up both going to Harvard.

**Gitschier:** Why did you want to work for Meselson rather than continue with Stahl?

**Ptashne:** Everybody agreed that Matt was a great intellect and a great figure, and besides, Frank, I’m sure, wouldn’t have had me after the tube mix-up.

**Gitschier:** So what year were you on the plane to Harvard?

**Ptashne:** Well, we’ve skipped Vietnam!

**Gitschier:** We have not skipped Vietnam. We haven’t even gotten to Harvard yet!

**Ptashne:** Oh that’s right, I graduated in ‘61. Yikes.

**Gitschier:** OK, so fall of ‘61, we’re going to Cambridge?

**Ptashne:** It *sounds* right. The main thing I remember [about graduate school] was dropping a huge vat of heavy water that Matt had obtained at great expense.

**Gitschier:** Oh, my God, and it broke.

**Ptashne:** Oh, did it break!

**Gitschier:** What was your thesis project on?

**Ptashne:** Ah, this I remember exactly!

**Gitschier:** Excellent.

**Ptashne:** Because I knew that the only reason to go into science was to solve the repressor thing. But, you couldn’t do that as a graduate student because the great minds of the world, including the French scientists and their international postdocs, had tried and failed.

So I had to do a warm-up on λ. It’s not worth going into detail now, but it had to do with an aspect of how λ’s viral chromosome attached to and came out of the host chromosome.

**Gitschier:** And you did your PhD work pretty quickly, right?

**Ptashne:** I guess so. But the idea was to put off getting the actual degree till later to avoid the draft. I became a Junior Fellow at the Harvard Society of Fellows.

**Gitschier:** OK, it’s ‘65-ish. Now you are a Junior Fellow, and you had done your warm-up experiments. What was it that made isolating λ repressor possible as a Junior Fellow, but not possible as a graduate student?

**Ptashne:** When I undertook to isolate the repressor, it was just at the time that Wally Gilbert had come from physics, and he was an assistant professor at the point.

**Gitschier:** Still in physics?

**Ptashne:** No, he switched under Jim Watson’s influence. He had been doing stuff with RNA and proving it was message.

So the reason I was a Junior Fellow was to do this repressor thing, and somehow it became clear that there was going to be a competition: me against Wally and Benno Müller-Hill, a postdoc in Wally’s lab. I, then, with Jim’s manipulation, hired Nancy Hopkins as my technician, and she turned out to be a real collaborator. Nowadays, her name would have been on the papers, but unfortunately, that was not the custom then for technicians. Wally and Benno were going after a putative repressor called the lac repressor, and Nancy and I were going after the putative λ repressor.

Then there was this big thing.

**Gitschier:** What big thing?

**Ptashne:** Ah, this huge race, 18 hours a day. It was even the subject of a made-for-TV film called “The Race for the Repressor.”

**Gitschier:** Did you start at basically the same time?

**Ptashne:** Yeah. And we used totally different ways of doing it.

**Gitschier:** Did you guys share information?

**Ptashne:** Yes. I have never had such a close scientific relationship with *anybody* as I had with Wally.

**Gitschier:** Even though you were competing. How is that?

**Ptashne:** It’s hard to explain, because it doesn’t seem possible. But that’s the way it was. Often we’d go home to Wally’s house, have dinner, and talk all evening. Some occasions were particularly fascinating because of an assembled larger family. The father of Wally’s wife Celia was Izzy [Isidor] Stone, and he and her mother produced a famous left-wing paper, *I*.*F*. *Stone’s Weekly*. Her brother-in-law was Leonard Boudin, a famous liberal lawyer.

Wally is an unusually intelligent person. In those days, we talked incessantly. I don’t know if he talked to anybody else, because he had a reputation for not saying much. And I think I learned more from Wally than anybody.

In the end, Wally and Benno published partial purification of the lac repressor a month before we published a corresponding paper on the λ repressor. We then discovered and published that λ repressor can bind to specific sites on DNA, and about a year later Wally and Benno showed that lac repressor can bind a different set of specific sequences. These experiments set an enormous field in motion.

As is usual in science, most of the time, it seemed that nothing is happening, but I clearly remember the day we discovered specific binding by λ repressor. I was sitting in a seminar and Nancy comes rushing into the seminar, waving a piece of paper and saying, “It worked, it worked!” So that showed it was a sequence-specific DNA binding protein.

**Gitschier:** So, going back to my question of why it was possible now to isolate the repressor, whereas it wasn’t earlier, I think what you are saying is that having this intense competition focused the mind and really challenged you—and Wally—to pull this off. One thing I’ve noticed in my own research career is that it is a lot easier to solve a problem once you know someone else thinks it’s solvable too.

**Ptashne:** As I recall, we were impressed at how easily the others seemed to give up. They were using traditional methods, and we had reason to think that those methods shouldn’t work because the repressors, whatever they were, were apt to be present in extremely small amounts per cell and because there was no enzymatic assay.

And so we hit on two different exotic assays designed to detect these molecules in concentrated solution full mostly of other things. For example, Nancy and I ended up with a preparation of λ repressor that was highly impure by standard assays, but which was preferentially radioactively labeled. For a while it came and went, until finally Nancy—and only Nancy—could inspect the bacterial colonies the night before and mysteriously pick out the ones that would work. Anyway, with the preferentially labeled stuff, and with the appropriate wild-type and mutant λ DNA molecules, it was feasible to ask whether, in a “velocity centrifugation experiment,” the repressor preferentially bound to the short DNA sequence called the operator. That’s the result I mentioned earlier that Nancy brought to the seminar.

**Gitschier:** At the outset, what was your thinking about how the repressors could work? Did you have a specific model in mind?

**Ptashne:** In their magnificent 1961 *JMB [Journal of Molecular Biology]* paper, Jacob and Monod had guessed that “the repressor” was RNA. This made sense because RNA can of course pair with a DNA strand of the corresponding sequence, but it was not at all obvious how a protein could do that. As I recall, even Francis Crick strongly doubted the possibility that proteins could do this. And if the protein could see the sequence, there were guesses that the DNA had to fold into a fancy structure that a typical protein could recognize. In the end, we tested—because we could—the simplest possible model, that λ repressor binds to specific sequences in normal double-stranded DNA. Thus the gradient experiment I just mentioned.

In the onslaught that followed, we and others showed that λ repressor can not only repress transcription of a gene, it can also work as an activator! For some time, the deep question was the mechanism of that activation. Did an activator confer some subtle change in the DNA helix that was transmitted to the gene, for example? I must say, I hated this idea because it was by then clear that in eukaryotes there were regulatory elements called enhancers that could activate genes positioned very far away (many thousands of base pairs) on the DNA. How could a transmission model explain that? And we refused to accept any model that couldn’t be generalized. One breakthrough was the design of genetic screens for λ repressor mutants that bind DNA normally but have lost the ability to activate transcription. Such mutants altered a surface on the repressor that we later called its “activating region.” Specific DNA binding could cause repression, but could not cause activation.

**Gitschier:** I ran across an introductory comment [In *Inspiring Science*: *Jim Watson and the Age of DNA*], “Ptashne’s successful search for, and characterization of, the elusive repressor of bacteriophage λ, work that spanned two decades, can fairly be regarded as the greatest sustained experiment of the last century.”

**Ptashne:** Joe Sambrook wrote that.

**Gitschier:** So one of the things that distinguishes you from many other scientists is that you really stuck with the λ problem, digging deeper and deeper into understanding the switch between lysogeny and lytic growth, and then went on to ask whether what you had learned from λ was applicable to higher organisms. Wally, for example moved on to other problems, cloning insulin, sequencing, etc. What compelled you to keep moving forward with such focus?

**Ptashne:** One great thing about explication of the λ switch is that, thanks to more and more inputs combining genetics, structural biology, etc., the system became ever more coherent. And so any finding had to be, and could be, explained. Although, in the early days, we were constantly surprised by discoveries of how the λ switch worked—for example, multiple operators, cooperative binding, positive control, a second protein [cro] that also recognized the λ operators—we were always able to fit these observations into a coherent picture that made very specific predictions, and after a while, when the predictions were mostly borne out, we felt that we really understood how things worked. Few biological systems are like that. In retrospect, this all depended on getting many seemingly minor details right!

And by the way, certain things became clear only later. For example, we knew that the switch could be subject to negative feedback, superimposed on the basic positive feedback, but only some years later did it emerge that there indeed was negative feedback—it depended on a “long-range” interaction between DNA-bound repressors and DNA looping. And the more we learn about gene regulation and development in higher organisms, the more it seems that λ teaches us.

Karl Popper said that basic models tell us more than we can at first know. This is tellingly apt as applied to the λ switch.

**Gitschier:** Let’s touch briefly on another one of your long-standing pursuits—the violin.

**Ptashne:** Well, I didn’t start early enough and I’m not good enough to be a professional violinist.

**Gitschier:** Why are you so passionate about it?

**Ptashne:** Perhaps a hint is a remark that Francis Crick made about how, when he switched into neurobiology, his colleagues were rather chirpier and more optimistic compared to his old friends. And the reason was that everyone accepted the fact that the problem was hopeless!

And playing the violin is just simply impossible, unless you’ve done it from the age of 5. On the other hand, if you work hard and have good teacher, every few weeks you can make a jump, and it’s like a religious experience. You can’t tell how it happened, but suddenly you can do something that you couldn’t do before. It is infinitely difficult, and it never ends. The other thing is that if you do anything seriously, you appreciate more and more what other humans accomplish, even if you can’t do it. I’m constantly filled with admiration for what these people do. If you don’t have the experience of struggling with it every day, you don’t know how hard it is.

And of course: the sound. Listen to Heifetz—Bach, Brahms, Sibelius, anything really. Thrilling, overwhelming, deeply moving. I set out on a long journey to somehow get a Guarneri del Gesu just like Heifetz’s! Turns out I’m no Heifetz, but then, nobody else is, either.

**Gitschier:** I want to close with a remark you made in an email to me. You alluded to something one of your former students [Bob Sauer, now at Massachusetts Institute of Technology] said about your philosophy of doing science. Because I think this relates to your skepticism about some of today’s approaches to science.

**Ptashne:** Sauer apparently said something I have long believed. There are various ways to put it, but it goes something like this:

I do not believe there are privileged ways to get answers, to solve problems in science. There are problems—often not so easy to formulate—but no sure path to answers. Scientists, for obvious reasons, tend to become expert in this or that—biochemistry or genetics, for example. And one can fall into the trap of thinking, “Aha, if I had an X-ray structure of something, I'd really understand!” Or, “If I had a mutant, I'd really understand!” Or, “If I could do physical chemistry and measure numbers, make models, etc., I'd really understand!”

Baloney. The great thing, as Sauer was pointing out I guess, is that we weren’t certified experts in anything. We conceptualized problems and did what was necessary to solve them, so to speak. And that attracted lots of different kinds of people into the lab. And so, over the years, we’ve had nascent biochemists (like Sauer), students of X-ray crystallography, and neophytic geneticists. Almost all were graduate students, at least half of them women. Serious, lively intellects. I love them all. Even Johnson.

